# Heath related quality of life and associated factors among adults with and without diabetes in Adama city East Shewa, Ethiopia 2019; using generalized structural equation modeling

**DOI:** 10.1186/s12955-020-01337-9

**Published:** 2020-03-30

**Authors:** Biruk Shalmeno Tusa, Bisrat Misganaw Geremew, Mekuriaw Alemayehu Tefera

**Affiliations:** 1 Department of Epidemiology & Biostatistics, Collage of Health and Medical Sciences, Haramaya University, Haramaya, Ethiopia; 2 Department of Epidemiology and Biostatistics, Institute of Public Health, College of Medicine and Health Sciences, University of Gondar, Gondar, Ethiopia; 3 Department of Environmental Occupational Health and safety, Institute of Public Health, College of Medicine and Health Sciences, University of Gondar, Gondar, Ethiopia

**Keywords:** Health-related quality of life, Diabetic mellitus, Generalized structural equation model

## Abstract

**Background:**

Diabetes mellitus (DM) is a chronic disease, leading to many complications and substantial decrease in patients’ Health Related Quality of Life (HRQoL). HRQoL among diabetic patients could affect by concurrent various factors. Therefore, analysis of these concomitant factors using generalized structural equation model (GSEM) that takes account the complex network of relationship could be a more utilitarian approach to better understand factor affecting HRQoL. The present study aimed to assesses the level of HRQoL and its associated factors among adults with and without diabetes.

**Methods:**

A comparative cross-sectional study was conducted from March 13 to April 4, 2019 in Adama Hospital and Medical College and Adama city Kebele 2, 4 and 5, East Shewa Ethiopia. Data related to socio-demographics, behavioral, clinical factors and HRQoL were collected from 359 adults with diabetes & 415 adults without diabetes through face to face interviews. Data was entered to Epi-data 3.1 then it was exported to STATA 14 for further analysis. GSEM was employed to verify relationships and association among exogenous, mediating and endogenous variable concurrently.

**Results:**

Diabetic groups had a significant lower mean score in all domains of HRQoL than non- diabetic group (*p*-value< 0.0001). Depression had a direct negative effect on all domains of HRQoL in both groups. Fasting blood sugar also had a direct negative effect on all domains of HRQoL except social relation. Diabetes mellitus complication had a direct negative effect on both physical and psychological health. Low Medication adherence and poor diabetic self-care activity had a negative direct, indirect and total effect on physical and environmental health through fasting blood sugar.

**Conclusion:**

Diabetic patients had lower HRQoL in all the domains of quality of life. Socio-demographic factor (Age, residence and marital status), clinical factor (Depression & Diabetes mellitus complication) and behavioral factor (diabetic self-care activity and medication adherence) mediated by fasting blood sugar were factor associated HRQoL among the diabetic group. Thus, we recommend that integration of screening for depression and give counseling on medication adherences and diabetic self-care activity along with the already existing DM treatment.

## Background

Diabetes mellitus (DM) is a group of metabolic disease characterized by hyperglycemia resulting from defects in insulin secretion, action or both [1]. It is one of the most common chronic diseases in nearly all counties and continues to increase in number and significance, as the changing life style leads to physical inactivity and increase obesity [2].

According to International Diabetic Federation (IDF) report, globally 424.9 million people were estimated to have diabetes in 2017 and are expected to rise above 628.6 million by 2045 [3]. The IDF also expects that until 2045 the number of adult patients suffering from diabetic in Africa will increase from 1.6 million to 4.6 million by 2045 [3]. In Ethiopia, the number of people with DM in 2017 was 2.6 million [3] with an annual average increase of DM was 5.4% [4].

Diabetes mellitus is a chronic disease, leading to many complication includes micro-vascular (nephropathy, retinopathy and neuropathy) and macro-vascular (stroke, coronary artery disease and diabetes foot ulcer) with co-morbidities lead to substantial decrease in patient’s quality of life (QoL) as well as socio-economical implication [5].

The WHO defines QoL as individuals’ perception of their position in life in which they live & in relation to their goals expectation, standard & concern. The definition considers individual satisfaction with their physical, psychological, social relationships and environment health. It also includes one facet on overall quality of life &general health [6].

In chronic diabetes patients, a complete cure cannot be attained rather clinical measures have provided for a good estimate of disease control with the ultimate goal of enhancing patient’s QOL [7]. Knowing the predictors and recognizing risk factors of QOL is essential and these factors may then be targeted for prevention [8]. QoL of patients with diabetic is an important factor for analysis of the effectiveness of medications and other care [5].

Some studies have been conducted on HRQoL and its associated factors among diabetic patients worldwide [5, 9–13] and in our Country Ethiopia [14–16]. However, those studies have demonstrated the relationships through univariate analysis. In that case the model could expect to determine the direct effect only from the independent variable of dependent variable, rather than the indirect effect. In addition, univariate analysis also has no ability to incorporate latent variables into the analysis.

In response to the above gaps, Generalized Structural equation model (GSEM) was employed for simultaneously analyze relationships among physical, environmental, social and psychosocial domain of HRQoL, the direct, and indirect effect of factors affecting HRQoL. In addition, adults without diabetes, was included as comparative group in order to compare the level of HRQoL between diabetic patient and health individuals. Therefore, the objective of the present study was to assess the level of HRQoL and its associated factors among adults with and without diabetes.

## Methods

### Study design and setting

Comparative-cross sectional study design was employed in Adama Hospital and Medical College (AHMC) among diabetic adults and Adama city among non-diabetic adults from March 13 to April 04, 2019. The Adama city is located 99 km southeast of Addis Ababa in the Great Rift Valley of East Africa on Ethio-Djibouti main road. In city there are 14 kebeles and AHMC is located in kebele 04. Currently, the hospital has a catchment population about 5million serving as a referral hospital for nearby hospital. The hospital runs several medical outpatient services including diabetic follow up clinic.

### Population

For the purpose of comparison two group’s population was recruited. Patients diagnosed with DM who had follow up for at least 6 month and age greater than 18 years and visit the facility (AHMC) during the study period were selected as study participants for group one. Non-diabetic individuals, age 18 years and above and resident of Kebele 02, 04 and 05 of Adama city were selected as study participants of group two after excluding those individuals who have other known chronic (self-reported chronic conditions) illness like Hypertension, asthma. Absence Symptoms of uncontrolled hyperglycemia (e.g. polyuria, polydipsia and polyphagia or a random (casual, non-fasting) plasma glucose concentration less than 200 mg/dL (11.1 mmol/L) were used as criteria for selection of non-diabetic patients.

### Sample size determination

A general rule of thumb is that the minimum sample size should be 5 ~ 20 times the number of parameters to be estimated [17]. In the diabetic group, we have 23 observed variables; the number of parameters to be estimated was 66. According to the foregoing rule, the minimum sample required in the diabetic group was 330 (5*66). In non-diabetic, we have 12 observed variables; the number of parameters to be estimated was 26. According to the foregoing rule, the minimum sample required in non-diabetic was 130 (5*26) then multiplying it by design effect 3 and it became 390. Therefore, the total sample size required for this study was 720.Then adding 10% non-response rate, then sample size became 792 (363 in diabetic group and 429 in non-diabetic).

### Sampling technique and procedures

A Systematic random sampling method was used to select Study participant. The sampling interval was calculated by dividing the expected number of diabetic patients per month into the sample size. The first study participant was selected by lottery method, and then the data were collected from each study participant with the interval of two until the desired sample size was reached.

Among 14 kebeles of Adama city, kebele 02 (Migra), 04 (Dhedecha arara) and 05(Degage) were selected by lottery methods for selection of eligible study participant for non-diabetic group. The estimated number of household in kebele 02, 04 and 05 are 4700, 6114 and 6110 households respectively. So we proportionally allocate our 429 non-diabetic group sample size for each kebeles (119 for kebele 02, 155 for kebele 04 and 05). The house-hold from each kebeles was selected by systematic random sampling. The sampling interval was determined by dividing the number of households in each kebeles by allocating sample size for each kebeles. For each selected household, one study participant was selected using the lottery method.

### Measurements

Data was collected through face to face interview with document review by clinical nurses and health extension worker after receiving training on how to collect the data using both semi-structured & standard questionnaire tools. The tools comprised of Socio-demographic characteristics, Behavioral (medication adherence, hazardous drinking habits and diabetic self-care activity), Clinical, depression symptoms related questions and the WHO-QoL tools.

Data related to socio-demographic and clinical factors were collected by using semi-structure and pre-tested questionnaire which was developed by first author. Variables such as treatment modality (oral hypoglycemic agent, insulin therapy, and both oral hypoglycemic and insulin), diabetes-related complications, fasting blood sugar (records from the last visits were taken) and presence of documented comorbidity were obtained from patients’ medical records.

Health related quality of life was measured using World Health Organization QoL Instrument (WHO-QoL -Brief) which is validated and widely used in Ethiopia [18]. WHO-QoL-BREF is a 26 item instrument consisting of four domains: physical health (7 items), psychological health (6 items), and the social relation (3 items), and environmental health (8 items), the overall perception of QoL and general health (2 items) [6].

Diabetes self-care practice was assessed using the 11-item Summary of Diabetes Self-Care Activities (SDSCA) scale [19] that was used and validated in previous studies in Ethiopia [20–22]. The SDSCA is a self-reporting measure of the frequency of performing diabetes self-care tasks, such as: - diet, exercise, blood glucose testing, and foot care over the preceding 7 days. To calculate the overall diabetic self-care practice value, we took the average of the mean values in each of the domains listed above. Study participants who scored equal to or above the mean in the SDSCA were classified as having good diabetes self-care practice and those who scored below the mean were considered as having poor self-care practice.

Medication adherence was measured using the standardized and widely utilized four-item Morisky Medication adherence scale (MMAS-4) [23]. It was used and validated in previous study in Ethiopia [22]. A high score indicates low levels of Medication adherence.

Hazardous drinking was assessed using Fast Alcohol Screening Test (FAST) that was used and validated in previous study in Ethiopia [24]. The item scores obtain from the scale were summed and hazardous drinking was considered when the score is 3 or more [25]. Depression symptoms were measured using the Kessler 6 scales [26] which is validated in Ethiopia [27]. A high score indicates high levels of depression.

Initially questionnaire was prepared in English version, then translated into Amharic and Oromiffa (local language) and again back translated to English by another person to check the consistence of the meaning.

### Data processing, model building and analysis

Each questionnaire was checked visually for completeness and consistency. Data was entered into the Epi-data 3.1 then it exported to STATA 14 for further analysis. Descriptive statistics and summary statistics were presented using text, figure and tables. Reliability was also be assessed for each domain of WHO-QoL –Brief using the Cronbach’s α coefficient and values of 0.7 or higher were considered satisfactory.

The score of each domain of WHO-QoL –Brief was obtained by summation of their corresponding items for each participant. Then the scores were transformed linearly to a 0–100-scale as described by authors [28]. We employed independent t-test to compare the mean score of domains between two groups (adults with and without diabetes) after checking normality and equal variance assumption.

Generalized Structural Equation Model (GSEM) was employed to examine relationships and prediction among socio-demographic factors, behavioral factors, clinical factors, Depression symptoms and HRQoL domains for each group. Each transformed domains score were continuous variable that was analyzed with Gaussian family and identity link function. Depression symptoms were latent variable which constitutes items with order response, their measurement model was analyzed with ordinal families with logit link function.

According to different literature DSA & medication adherence have an effect on FBS. Not only that, these factors also had an effect on domain of HRQoL. By considering the above issue we plan to make FBS as mediator variable in order to assess direct and indirect effect of DSA and medication adherence on domain of HRQoL through FBS. So, fasting blood sugar was continuous variable that was analyzed with Gaussian family and identity link function.

**Fig. 1:**
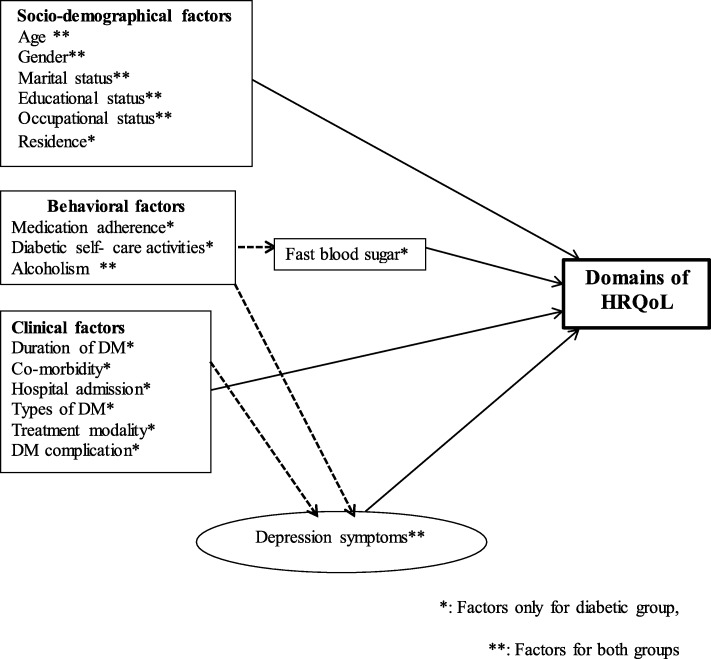
Hypothesized model for HRQoL and associated factors among Adults with and without diabetes in Adama city, 2019. *: Factors only for diabetic group,**: Factors for both groups

We started the analysis with a hypothesized model in Fig. 1 for each group. Modification were taken iteratively by adding a path link or including mediating variable (in group one). Finally, an over identified model with minimum information criteria was retained. A final model was selected based on statistical significance of path coefficient, the theoretical meaningfulness of the relationship and minimum information criteria.

Diagrammatically, the effect of each exogenous or mediating variable on the respective dependent variable was indicated by the path coefficient along with a single headed arrow, and the correlation between measurement errors (residual errors that reflect the unexplained variation in the observable endogenous variables due to all unmeasured causes) was indicated by double arrows. Statistically significant effects were assumed for *P* <  0.05 at Confidence interval of 95%.

## Results

### Socio-demographical, behavioral and clinical characteristics

A total of 774 study participants (359 adults with DM and 415 adults without DM) were included in a study with a response rate of 97.72%. The Socio-demographical, behavioral and clinical characteristics of two groups are presented in Table 1. Table 1 Socio-demographical, Behavioral and clinical characteristics of adults with and without DM in Adama Adults with diabetes were more likely to be uneducated, retired and non-Hazardous drinker than their counterparts. The mean depression symptom scale score was higher for adults with DM than adults without DM (*p*-value < 0. 0001).

**Table 1:** Socio-demographical, Behavioral and clinical characteristics of adults with and without DM in Adama city

Variable	Diabetic (***n*** = 359)	Non-diabetic (***n*** = 415)	***P***-value
**AGE**; Mean (SD)	51.64(14.51)	33.37(9.86)	< 0.0001*
**Depression** Mean (SD)	2.35(0.03)	1.97 (0.03)	< 0.0001*
**Gender**; n (%)			
Female	176(49.03)	259(62.41)	< 0.0001**
Male	183(50.97)	156(37.59)	
**Residence**; n (%)			
Urban	306(85.24)	415(100)	
Rural	53(14.76)	–	
**Educational status**; n (%)			
Uneducated	60(16.71)	34(8.25))	< 0.0001**
Primary cycle	119(33.15)	104(25.24)	
Secondary and above	180(50.14)	274(66.50)	
**Marital status**; n (%)			
Single	45(12.53)	134(32.29)	< 0.0001**
Married	225(62.67)	228(54.94)	
Separated	15(4.18)	13(3.13)	
Divorced	10(2.79)	16(3.86)	
Widowed	64(17.83)	24(5.78)	
**Religion**			
Christen	271(75.49)	292(70.36)	< 0.0001***
Muslim	87(24.23)	105(25.30)	
Other	1(0.28)	18(4.34)	
**Occupational status**; n (%)			
Employed	76(21.17)	165(39.76)	
Housewives	103(28.69)	69(16.63)	
Retired	71(9.95)	6(1.45)	
Merchant	35(9.75)	61(14.70)	
Daily labor	7(1.95)	43(10.36)	
Jobless	35(9.75)	51(12.29)	
Farmer	32(8.91)	3(0.72)	
Other	0(0)	17(2.20))	
**Hazardous drinking; n (%)**			
Yes	22(6.13)	64(15.42)	< 0.0001**
No	337(93.87)	351(84.58)	

* *P*-values from two-sample t test with equal variances; **p-values from Chi-square test; ***p-values from Fisher exact test

**Fig. 2:**
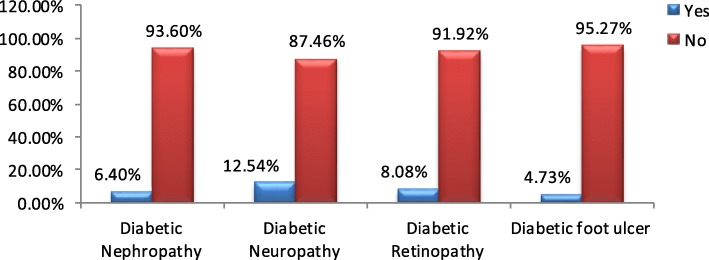
Shows Prevalence of Diabetic related complication of adults with DM attending at AHMC from March 13 to April 04, 2019

Of 359 Diabetic study participants, 186 (51.96%) had poor diabetic self-care practice 101 (28.13%) of them have low medication adherence and 83 (23.12%) of them had developed diabetic related complication (Fig. 2). Mean fasting blood sugar level was 151.07(SD = 38.21) mg/dl in diabetic group.

### Internal consistency & correlations between the domains of the WHO-QoL –brief

To check the internal consistency, the Cronbach’s alpha was calculated for each domain of the instrument. All domains of WHO-QoL –Brief had high values of Cronbach’s alpha (α > 0.7). Inter domain correlation showed that, there were statistically significant correlation between domains. The correlation was weak between physical health & social relation as compared to others (Table 2).

**Table 2:** Internal consistency & Correlations between the domains of the WHO-QoL –Brief

Domains	Cronbach’s alpha	PH	PsyH	SR	EH
**PH**	0.90	1.00			
**PsyH**	0.83	0.76*	1.00		
**SR**	0.79	0.50*	0.53*	1.00	
**EH**	0.78	0.55*	0.65*	0.51*	1.00

*PH* Physical health, *PsyH* Psychological health, *SR* Social relation, *EH* Environment Health

*Correlation is significant at the 0.05 level (2-tailed)

### Comparison HRQoL among adults with and without DM

Adults with DM had a significant lower mean score in Overall perception of QoL, General health & all domains of Health related quality indicating poor quality of life. The difference was large in Physical Health (28.64) and small in Environmental Health (8.99) domains (Table 3).

**Table 3:** Comparison of HRQoL among adults with and without DM in Adama city, 2019

Domain of HRQOL & Overall QoL	Diabetic (***n*** = 359) Mean (SD)	Non-diabetic (***n*** = 415) Mean (SD)	Mean difference	***p***-value
**Overall QoL**	43.25(19.90)	65.48(23.88)	22.24	< 0.0001*
**General health**	42.27 (17.56)	76.45(27.60)	24.30	< 0.0001*
**Physical health**	45.40(15.38)	74.04(17.63)	28.64	< 0.0001*
**Psychological health**	53.99(10.35)	75.85(15.75)	21.86	< 0.0001*
**Social relation**	53.86(20.92)	73.89(21.65)	20.02	< 0.0001**
**Environmental health**	48.13(9.87)	58.34(11.55)	8.99	< 0.0001*

*p-values from two-sample t-test with unequal variances

**p-values from two-sample t-test with equal variances

### Factors associated with health related quality of life

**Fig. 3:**
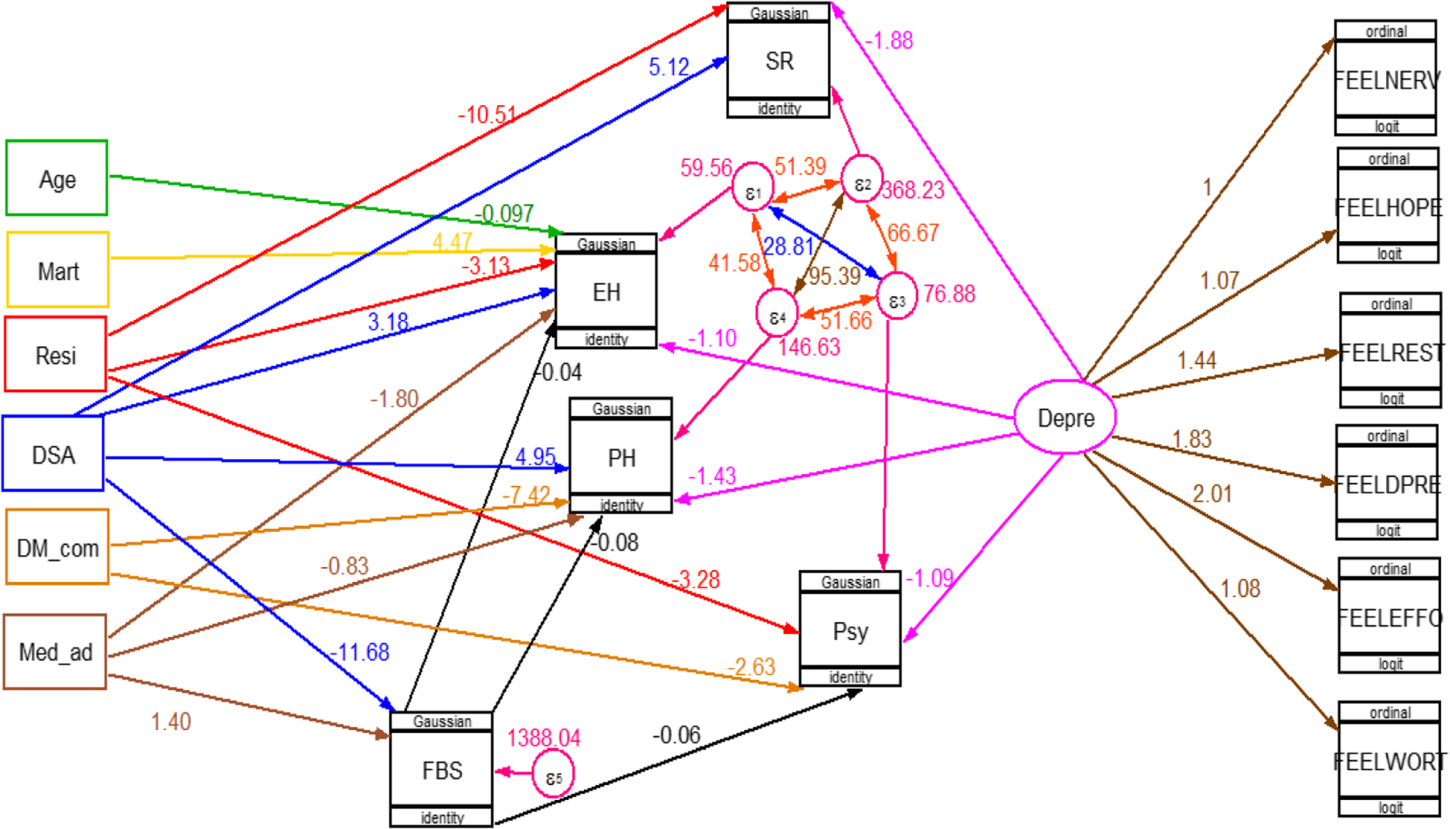
GSEM for factor associated with HRQoL among of adults with DM attending at AHMC from March 13 to April 04, 2019. Mart: Marital status, Resi: Residence, Med_ad: Medication Adherence, DSA: diabetic self-care activity, FBS: Fasting blood Sugar, DM_com: DM complication, Depre: Depression, PH: Physical health, Psy: Psychological health, SR: Social relation &EH: Environment Health] Single headed arrows show direction of effect; double headed arrow shows correlation

**Fig. 4:**
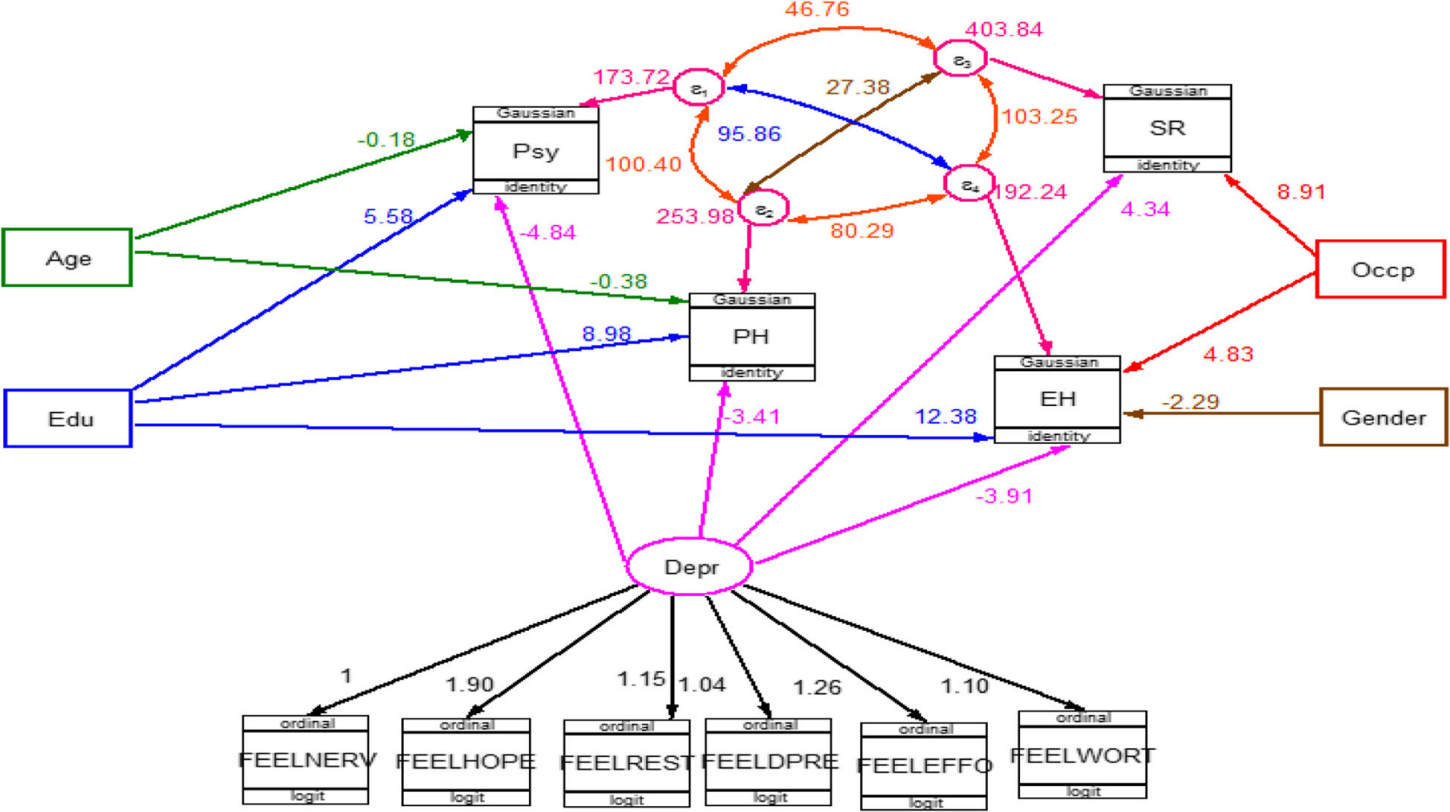
GSEM for factor associated with HRQoL among of adults without DM in Adama city from March 13 to April 04, 2019. Edu: Educational status, Occp: Occupational status Depre: Depression, PH: Physical health, Psy: Psychological health, SR: Social relation &EH: Environment Health] Single headed arrows show direction of effect; double headed arrow shows correlation

The final model containing both structural component (relationships among latent or observable variables) and measurement component (relationships among latent variables and its items) for group one (adults with DM) and for group two (adults without DM) is shown in Figs. 3 and 4 respectively. In each figure all the path coefficients were statistically significant at an alpha level of 0.05.

### Factors associated with HRQoL of life among adults with DM

In group one (adults with DM) several variables, namely gender, educational status, occupational status, type of DM, duration of DM, drug regimen, Co-morbidities, Hospital admission, smoking and hazardous drinking were excluded from the final model as their contribution were not statistically significant at an alpha level of 0.05.

As we can see from Fig. 3 model for group one (adults with DM) had included seven exogenous variables (age, marital status, residence, medication adherence, diabetic self-care activity, DM complication and depression), one mediator variables (fasting blood sugar) and four endogenous (Physical health, Psychological health, Social relation &Environment Health). Two exogenous variables, namely medication adherence and diabetic self-care activity were both directly and indirectly related with physical, psychological and environmental heath domain’s HRQoL via mediating variable fasting blood sugar.

Being rural residents had a direct negative effect on psychological health (β = − 3.28, 95% CI − 5.58 to − 0.98), Social relation (β = − 10.51, 95% CI − 15.80 to − 5.22) and Environment Health (β = − 3.13, 95% CI − 5.28 to − 0.98). Older age & Being married had a direct negative effect (β = − 0.10, 95% CI − 0.17 to − 0.03) and a direct positive effect (β = 4.47, 95% CI 1.76–7.17) on Environment Health domains respectively.

Increased fasting blood sugar level had a direct negative effect on physical health (β = − 0.08, 95% CI − 0.11 to − 0.47), psychological health (β = − 0.06, 95% CI − 0.09 to − 0.04) and environmental health (β = − 0.04, 95% -0.06 to − 0.01) domains.

Being live with DM complications had a direct negative effect on both physical health (β = − 7.42, 95% CI − 10.15 to − 4.69) and psychological health (β = − 2.63, 95% CI − 4.62 to − 0.63) domains. Depression had a direct negative effect on all domains of HRQoL, on physical health (β = − 1.43, 95% CI − 1.96 to − 0.89), on psychological health (β = − 1.09, 95% CI − 1.47 to − 0.70), on the social relation (β = − 1.88, 95% -2.66 to − 1.11) and environmental health (β = − 1.10, 95% -1.47 to − 0.74).

Among upstream variables (exogenous variable related to the mediator variable, fasting blood sugar), low medication adherence had both a negative direct (β = − 2.95, 95% CI − 5.65 to − 0.26) and indirect (β = − 0.98, 95% CI − 10.13 to − 0.33) effect on physical health that resulted in a total negative effect (β = − 3.93, 95% CI − 15.78 to − 0.59). Similarly Good diabetic self-care activity had both a positive direct (β = 4.95, 95% CI 2.66 to 7.25) and indirect (β = 0.93, 95% CI 1.81 to2.15) effect, accordingly a total positive effect (β = 5.88, 95% CI 4.47 to 9.40) on physical health.

Good diabetic self-care active had only a direct positive (β = 5.12, 95% CI 1.29 to 8.96) effect on social relations. However, it had both a positive direct (β = 3.18, 95% CI 1.62 to 4.73) and indirect (β = 0.47, 95% CI 0.04 to 1.17) effect consequently a total positive effect (β = 3.65, 95% CI 1.56 to 5.90) on environmental health.

Low medication adherence had both a negative direct (β = − 2.69, 95% CI − 4.52 to − 0.86) and indirect (β = − 0.49, 95% CI − 0.22 to − 0.17) effect on environmental health that lead to a total negative effect (β = − 3.18, 95% CI − 4.74 to − 1.03).

Low medication adherence and good diabetic self-care activity had a direct positive effect (β = 12.22, 95% CI 2.90 to 21.56) and a direct negative effect (β = − 11.68, 95% CI − 19.54 to − 3.83) on fasting blood respectively. (Table 4).

**Table 4:** The Direct, Indirect and Total effect of socio-demographical, clinical and Behavioral factor on HRQoL domains among of adults with DM attending at AHMC, 2019

Variables (effect measure is β) Diabetic (***n*** = 359)	Direct effect (95% CI)	Indirect effect (95% CI)	Total effect (95% CI)
**DV: Physical health**			
Fasting blood sugar	− 0.08[− 0.11 to − 0.47]	–	–
Depression	**−** 1.43[− 1.96 to − 0.89]	–	–
DM complication			
No	0	0	0
Yes	−7.42[− 10.15 to − 4.69]	–	–
Medication Adherence			
High	0	0	0
Medium	− 0.83[− 3.50 to 1.84]	–	
Low	− 2.95[− 5.65 to − 0.26]	−0.98[− 10.13 to − 0.33]	− 3.93[− 15.78 to − 0.59]
Diabetic self-care activity			
Poor	0	0	0
Good	4.95[2.66–7.25]	0.93 [1.81 to 2.15]	5.88[4.47 to 9.40]
**DV: Psychological health**			
Residence			
Urban	0	0	0
Rural	−3.28[− 5.58 to −0.98]	–	–
Fasting blood sugar	−0.06[− 0.09 to − 0.04]	–	–
Depression	− 1.09[−1.47 to − 0.70]	–	–
DM complication			
No	0	0	0
Yes	−2.63[− 4.62 to −0.63]	–	–
**DV: Social relation**			
Residence			
Urban	0	0	0
Rural	−10.51[−15.80 to −5.22]	–	–
Depression	−1.88[−2.66 to − 1.11]	–	–
Diabetic self-care activity			
Poor	0	0	0
Good	5.12[1.29–8.96]	–	–
**DV: Environmental health**			
Age	−0.10[−0.17 to −0.03]	–	–
Residence			
Urban	0	0	0
Rural	−3.13[−5.28 to −0.98]	–	–
Marital status			
Single	0	0	0
Married	4.47[1.76–7.17]	–	–
Separated	3.76[−0.55 to 8.07]	–	–
Divorced	8.29[3.44–13.10]	–	–
Widowed	3.24[−0.37 to 6.86]		
**DV: Environmental health**			
Depression	−1.10[−1.47 to −0.74]	–	–
Fasting blood sugar	−0.04[− 0.06 to − 0.01]	–	–
Medication Adherence			
High	0	0	0
Medium	−1.80[−3.60 to −0.01]	–	–
Low	−2.69 [−4.52 to − 0.86]	−0.49[− 0.22 to − 0.17]	−3.18[− 4.74 to − 1.03]
Diabetic self-care activity			
Poor	0	0	0
Good	3.18[1.62–4.73]	0.47[0.04 to 1.17]	3.65[1.56 to 5.90]
**DV: Fasting blood sugar**			
Medication Adherence			
High	0	0	0
Medium	1.40[−8.29 to 11.10]	–	–
Low	12.22[2.90 to 21.56]	–	–
Diabetic self-care activity			
Poor	0	0	0
Good	−11.68[−19.54 to −3.83]	–	–

*DV* dependent variable, *CI* confidence interval

### Factors associated with HRQoL of life among adults without DM

In group two (adults without DM), hazardous drinking and marital status excluded from the final model as their contribution were not statistically significant at an alpha level of 0.05. As we can see from Fig. 4 the final model for non-diabetic group had included five exogenous variables (age, gender, educational status, occupational status and depression) and four endogenous (Physical health, Psychological health, Social relation & Environment Health).

Older age had a direct negative effect on both physical health (β = − 0.38, 95% CI − 0.54 to − 0.22) and psychological health (β = − 0.18, 95% CI − 0.31 to − 0.60) domains. Being employed had a direct positive effect on social relations (β = 8.91, 95% CI 2.21 to 15.61) and environmental health (β = 4.83, 95% CI 0.82 to 8.84) domains.

Male in gender had a direct negative effect (β = − 2.96, 95% CI − 5.36 to − 0.56), on environmental health domain of HRQoL. Being educated also had a direct positive effect on physical, psychological and environmental health domains. The strength of the effect was stronger for those who attend secondary cycle than primary cycle (Table 5).

**Table 5:** The Direct effect of socio-demographical, clinical and Behavioral factors on HRQoL domains among of adults without DM in Adama city from March 13 to April 04, 2019(Derived from GSEM)

Variables Non-diabetic (***n*** = 415)	Health related quality of life domains (effect measure is β)			
	Physical health	Psychological health	Social relation	Environmental health
**Age**	−0.38[−0.54 to 0.22]	−0.18[− 0.31 to − 0.60]	–	–
**Gender**				
Female	0	0	0	0
Male	–	–	–	−2.96[−5.36 to −0.56]
**Educational status**				
Uneducated	0	0	0	0
Primary cycle	8.98[2.69–15.28]	5.58[0.37–10.79]		12.38[7.13–17.62]
Secondary & above	9.06[3.11–15.27]	9.16[4.24–14.08]		17.68[12.55–22.81]
**Occupational status**				
Jobless	0	0	0	0
Housewives	–	–	3.31 [−4.28 to 10.90]	3.58[−1.11 to 8.27]
Retired	–	–	8.06 [−9.08 to 25.20]	6.12[− 4.41 to 16.62]
Merchant	–	–	6.48[−1.31 to14.27]	4.74[0.01 to 9.47]
Daily labor	–	–	5.53 [−2.86 to 13.93]	3.58[−1.11 to 8.27]
Employed	–	–	8.91[2.21 to 15.61]	4.83[0.82 to 8.84]
Farmer	–	–	21.10[−2.16 to 44.3]	10.26[−3.77 to 24.30]
Students	–	–	−6.55[−22.39 to 9.30]	9.33[−0.02 to18.68]
**Depression**	− 3.41[−4.75 to − 2.06]	−4.84[−6.21 to-3.47]	−4.34[− 6.04 to − 2.64]	− 3.91[−5.18 to − 2.64]

*CI* confidence interval

Similar to diabetic group, depression also had a direct negative effect on all domains of HRQoL in non-diabetic. On the physical health (β = − 3.41, 95% CI − 4.75 to − 2.06), on psychological health (β = − 4.84, 95% CI − 6.21 to − 3.47), on the social relation (β = − 4.34, 95% -6.04 to − 2.64) and environmental health (β = − 3.91, 95% -5.18 to − 2.64).

### Comparison between two models

In this study, we compare the HRQoL of adults with and without DM. This study also assesses the effect of socio-demographical, clinical and behavioral factors on HRQoL using a Generalized Structural Equation model for each separated groups.

In both model, age & depression were included in the final model as their contribution were significant at alpha level 0.05. More specifically high level depression symptom were associated lower HRQoL across all domain in both model.

In model (diabetic group), additional variable related to their DM status (medication adherence, diabetic self-care activity, DM complication and fasting blood sugar) were included in the final model.

## Discussions

### Comparison of HRQoL among adults with &without DM

According to our study, Adults with DM were found to have lower quality of life in all domains of the WHOQOL-BREF and in overall quality life than adults without DM. This finding is congruent with other pervious comparative studies [9–11, 13, 29]. This consistence result might be, Diabetes often leads to the development of physical disabilities that, in turn, can have a detrimental effect on a patient’s quality of life [30].

The mean score difference among diabetic and non-diabetic on Environmental health was smaller. There is a study [9] which had steady finding with our study. This implied that bad environmental condition affects HRQOL in similar fashion in both groups.

Our finding demonstrated that, among domains of HRQoL, the physical health was the most affected domain in diabetic group and this is of course in agreement with other reports [14, 16, 31]. This consistency could be defensible by diabetes has more physical than mental (psychological) manifestations [32]. This could also be described by patients with diabetes, having higher degrees of complications like diabetic foot, which can disturb their physical ability to do day-to-day activities.

According to our study, psychological health and social relation was the least affected domain among the diabetic group. This finding is in line with other studies that was conducted southwest [15] and northwest [14] Ethiopia and other countries [5, 9, 31]. This consistence result in social relation and psychological health might be their social-culture that gives support for diseased individuals & diabetic patients manifest more physically than mentally (psychologically) respectively [32].

### Factor associated with HRQoL in diabetic patients

The current study shows that an older age was associated with poor environmental health in diabetic group. This finding is lined with previous studies conducted in different research setting [9, 12, 16, 33, 34]. Such outcome may indicate that younger people are more likely to adore better health than elder and this may not surprising because as age increase the physiological function decline and prevent different activity of the body which might impair HRQOL .

A study conducted in refugee camps in the Gaza strip [9] documented that male adults have lower HRQoL in social relation domain than female adult. However the present study demonstrates that gender have no effect on any domain of HRQoL. This discrepancy result might be due to difference of their study participant.

In our study in diabetic group, being rural resident was associated with lower HRQoL for a social relation domain. There is a study [23] which had consistence finding with our study. This congruent finding might be rural dwellers more likely uneducated and have the low idea about diabetes mellitus. Because of that, rural dwellers might not give support for diabetic patients and also diabetic patients have a low social relationship with their community.

Being married is another socio-demographic factor that had a positive effect on environmental health among diabetic groups and this is of course in congruent with other reports [28, 29]. This positive effect is due to, support from others can facilitate recovery from physical illness and enhance the ability to cope with and adapt to the consequences of chronic illnesses.

The present study showed that high level depression symptoms were associated with lower HRQoL across all domains in diabetic group. Different scholars also documented that co-occurrence of diabetes mellitus and depression symptoms decrease HRQoL [15, 35–37]. This consistence finding might be the effects of depression on health related quality life are comparable with those of arthritis, diabetes, and hypertension and Comorbid depression can exacerbate the effects of medical illness and may be an independent source of suffering and disability [38].

Depressive symptoms may also even forecast the occurrence of functional limitations and jeopardize the ability of diabetic individuals to take care of themselves [36]. Therefore, increased alertness for depression in diabetes care is needed. This can be accomplished by comprising screening tools for depression as portion of regular diabetes care.

The current study suggested that, presence of diabetic complication was one of the clinical factor that worth HRQoL for both physical and psychological health among diabetic patients. This finding is in line with previous studies that were conducted at different research setting [34, 39, 40]. This worth in HRQoL among diabetic who develop a complication might be due to, they need a considered amount of time for managing complications on the clinic and hospital admission. More specifically, those who develop diabetic foot ulcer have anxiety to possible of amputation [33] which might result impairing of HRQOL of DM patients.

According to this study, fasting blood sugar had an inverse association with all domains of health related quality of life except social relation. This finding is in line with the study that was conducted by Gebremedhin T et al. [16]. This consistence could be due to high blood glucose manifesting as like polyuria (excessive urination), polydipsia (excessive thirsty), polyphagia (excessive hunger), general weakness and sleeping disturbances [41], which may impair HRQoL. This can also be defensible as those who have higher blood glucose need more health care services, are powerless to perform their daily activities and are incompetent to join in different activities, paying to impair HRQoL.

According to the finding of current study, diabetic self-care activity had both a positive direct and indirect effect that resulted in a total positive effect on physical health. As per our knowledge only one study has investigated the HRQoL in adults with diabetes using structural equation model [42] and found similar results.

As mediator variable, fasting blood sugar was associated with poor medication adherence and diabetic self-care activity. According to the current study low medication was associated with increased fasting blood sugar level and this is of course coherent with other report [43]. Similarly, poor diabetic self-care activity was associated with increased fasting blood sugar level and this is of course coherent with other report [15, 44].

### Factor associated with HRQoL in non-diabetic individuals

The current study showed that male adults had lower health related quality life in environmental health domains than female adults among non-diabetic group and this is of course in congruent with another report [9]. This consistence result might be male adults might be more like to be addicted to alcohol and cigarettes than their counterparts and this leads to impaired health related quality of life.

Study conducted in Spanish among healthy adults, documented that HRQoL declines with decreasing educational level [45]. Similarly the present study demonstrated that being educated had a direct positive effect on physical, psychological and environmental health domains among non-diabetic groups. The strength of effect was stronger for those who attend secondary cycle than primary cycle.

This consistence finding might be educational level is personal variable which is in close relation with the ability of the person to manage environmental risks (exposure to threatening living situation, lack of stimulation and inappropriate nutrition) [46]. Education is also one of the most important factors affecting physical and cognitive function, and influences the quality of life indirectly, through achieving knowledge and expertise, life habits, job situations and income levels [47].

### Strength and limitation

The main strength of current study is, it used multivariate analysis (GSEM) to assess the effect socio-demographical, clinical and behavioral factors on health related quality of life. This enables to incorporate latent variable like depression and to determine direct, indirect and total effect when mediation effect present which has not been addressed by other studies. In addition to check whether there is a significant differences in the level Health related quality of life, the current study used non diabetic adults as comparative group.

However, the finding of this study interpreted with some limitation. The data were collected through face to face interview by considering the different educational level of respondents and this might prone to social desirability bias and could overestimate the result. In the tool there were questions that ask about their life in the last 4 weeks. These might prone to recall bias and could over or under estimate the result. An abundant of factors such as loss of families due to death, trauma and un-diagnosed other chronic illness can have an impact on HRQoL of the participants included in this study.

Moreover, the present study has been conducted in single center (AHMC) which limits the generalizability of the finding in Ethiopia; further multicenter studies are needed to address this issue.

## Conclusions

Diabetes adults had poor quality of life in all domains of WHOQOL-BREF and overall quality of life as compared with adults without diabetes. Socio-demographic factors (Age, residence and marital status), clinical factor (Depression &DM complication) and behavioral factors diabetic self-care activity and medication adherence) mediated by fasting blood sugar were factors associated with HRQoL among the diabetic group. Socio-demographic factors (Age, gender, educational status and occupation status), clinical factor (Depression) were factors associated with HRQoL among non-diabetic group. Thus, we recommend that integration of screening for depression and give counseling on medication adherences and diabetic self-care activity along with the already existing DM treatment.

## Data Availability

The datasets supporting the conclusions of this article are available upon request to the corresponding author.

## Abbreviations

AHMC: Adama Hospital and Medical College
DM: Diabetic Mellitus
FAST: Fast Alcohol Screening Test
GSEM: General Structural Equation Model
HRQoL: Health Related Quality of Life
IDF: International Diabetic Federation
MMAS: Morisky Medication Adherence Scale
QoL: Quality of Life
SD: Standard Deviation
SDSCA: Summary of Diabetes Self-Care Activities
WHO: World Health Organization
